# Seventy-Five Trials and Eleven Systematic Reviews a Day: How Will We Ever Keep Up?

**DOI:** 10.1371/journal.pmed.1000326

**Published:** 2010-09-21

**Authors:** Hilda Bastian, Paul Glasziou, Iain Chalmers

**Affiliations:** 1 German Institute for Quality and Efficiency in Health Care (IQWiG), Cologne, Germany; 2 Centre for Research in Evidence-Based Practice, Faculty of Health Sciences, Bond University, Gold Coast, Australia; 3 James Lind Library, James Lind Initiative, Oxford, United Kingdom

## Abstract

Hilda Bastian and colleagues examine the extent to which critical summaries of clinical trials can be used by health professionals and the public.

**Summary Points**

- When Archie Cochrane reproached the medical profession for not having critical summaries of all randomised controlled trials, about 14 reports of trials were being published per day. There are now 75 trials, and 11 systematic reviews of trials, per day and a plateau in growth has not yet been reached.
- Although trials, reviews, and health technology assessments have undoubtedly had major impacts, the staple of medical literature synthesis remains the non-systematic narrative review. Only a small minority of trial reports are being analysed in up-to-date systematic reviews. Given the constraints, Archie Cochrane's vision will not be achieved without some serious changes in course.
- To meet the needs of patients, clinicians, and policymakers, unnecessary trials need to be reduced, and systematic reviews need to be prioritised. Streamlining and innovation in methods of systematic reviewing are necessary to enable valid answers to be found for most patient questions. Finally, clinicians and patients require open access to these important resources.

Thirty years ago, and a quarter of a century after randomised trials had become widely accepted, Archie Cochrane reproached the medical profession for not having managed to organise a “critical summary, by speciality or subspeciality, adapted periodically, of all relevant randomised controlled trials” [1]. Thirty years after Cochrane's reproach we feel it is timely to consider the extent to which health professionals, the public and policymakers could now use “critical summaries” of trials for their decision-making.

## The Landscape

Keeping up with information in health care has never been easy. Even in 1753, when James Lind published his landmark review of what was then known about scurvy, he needed to point out that “… before the subject could be set in a clear and proper light, it was necessary to remove a great deal of rubbish” [2]. And 20 years later, Andrew Duncan launched a publication summarising research for clinicians, lamenting that critical information “…is scattered through a great number of volumes, many of which are so expensive, that they can be purchased for the libraries of public societies only, or of very wealthy individuals” [3]. We continue to live with these two problems—an overload of unfiltered information and lack of open access to information relevant to the well-being of patients.

**Figure 1:**
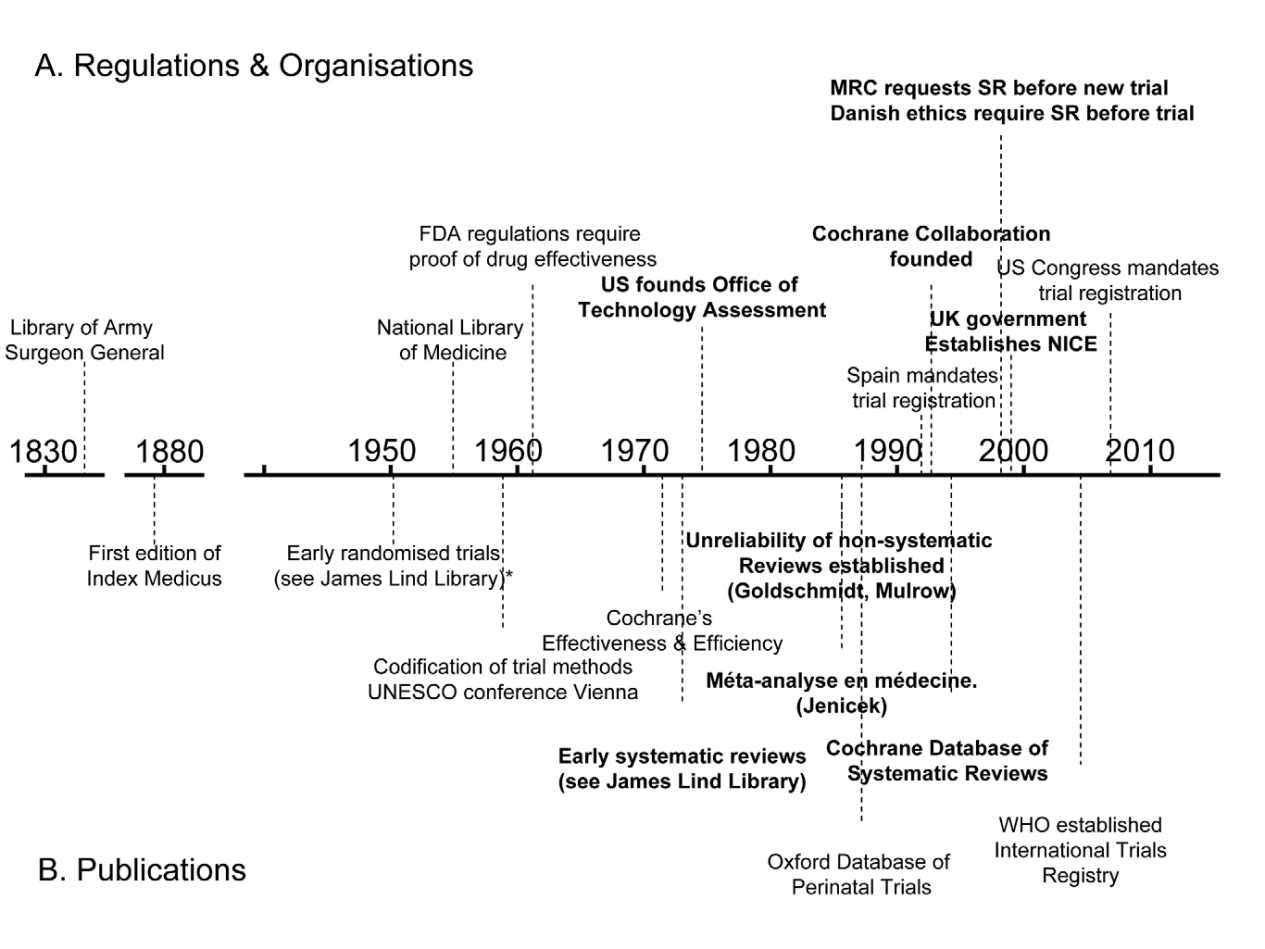
Policy and academic milestones in the development of trials and the science of reviewing trials.

A century later, the precursor of the US National Library of Medicine (NLM) began indexing the medical literature. Between 1865 and 2006, the index grew from 1,600 references to nearly 10 million [4]. Even with the assistance of electronic databases such as NLM's MEDLINE, the problem of having to trawl through and sift vast amounts of data has grown. As mountains of unsynthesised research evidence accumulate, we need to keep improving our methods for gathering, filtering, and synthesising it. Some of the key events in the story so far are shown on the timeline in Figure 1.

A legal regulatory framework overseen by the US Food and Drug Administration (FDA) requiring proof of efficacy of new drugs was introduced in 1962, and other countries followed suit. These developments made it inevitable that randomised trials would increasingly become an important component of the evidence base [5]. Government health technology assessment agencies were also established as policymakers sought to have more reliable evidence of the effects of other forms of health care interventions [6].

As the number of clinical trials grew, so too did the science of reviewing trials. Systematic reviews and meta-analyses endeavouring to make sense of multiple trials began to appear in a variety of health fields in the 1970s and 1980s (see Box 1). An important early example showed that postoperative radiotherapy after surgical treatment of breast cancer was associated with a previously unrecognised increased risk of death [7]. Another challenged beliefs about vitamin C and the common cold [8]. A third suggested a previously unrecognised advantage of some forms of fetal monitoring during labour in reducing neonatal seizures [9].

**Box 1. Early Systematic Reviews of the Effects of Health Care Interventions**

- Stjernswärd J (1974) Decreased survival related to irradiation postoperatively in early breast cancer. Lancet 304: 1285-1286.
- Chalmers TC (1975) Effects of ascorbic acid on the common cold. An evaluation of the evidence. Am J Med 58: 532-536.
- Cochran WG, Diaconis P, Donner AP, Hoaglin DC, O'Connor NE, Peterson OL, Rosenoer VM (1977) Experiments in surgical treatments of duodenal ulcer. In: Bunker JP, Barnes BA, Mosteller F, eds. Costs, risks and benefits of surgery. Oxford: Oxford University Press. pp 176-197.
- Smith ML, Glass GV (1977) Meta-analysis of psychotherapy outcome studies. Am Psychol 32: 752-760.
- Hemminki E, Starfield B (1978) Routine administration of iron and vitamins during pregnancy: Review of controlled clinical trials. Br J Obstet Gynaecol 85: 404-410.
- Hemminki E, Starfield B (1978) Prevention and treatment of premature labour by drugs: Review of controlled clinical trials. Br J Obstet Gynaecol 85: 411-417.
- Chalmers I (1979) Randomized controlled trials of fetal monitoring, 1973–1977. In: Thalhammer O, Baumgarten K, Pollak A, eds. Perinatal medicine. Stuttgart: Georg Thieme. pp 260-265.
- Policy Research Incorporated (1979) Medical Practice Information Demonstration Project. Bipolar disorder, a state of the science report. Baltimore: Policy Research Incorporated.
- Editorial (1980) Aspirin after myocardial infarction. Lancet 1:1172-1173. [Published anonymously but written by Richard Peto.]
- Baum ML, Anish DS, Chalmers TC, Sacks HS, Smith H, Fagerstrom RM (1981) A survey of clinical trials of antibiotic prophylaxis in colon surgery: Evidence against further use of no-treatment controls. N Engl J Med 305:795-799.
- Hampton JR (1982) Should every survivor of a heart attack be given a beta-blocker? Part I: Evidence from clinical trials. BMJ 285:33-36.
- Stampfer MJ, Goldhaber SZ, Yusuf S, Peto R, Hennekens CH (1982) Effect of intravenous streptokinase on acute myocardial infarction: Pooled results from randomized trials. N Engl J Med 307: 1180-1182.
- Sacks HS, Chalmers TC, Berk AA, Reitman D (1985) Should mild hypertension be treated? An attempted meta-analysis of the clinical trials. Mt Sinai J Med 52: 265-270.
- Yusuf S, Peto R, Lewis J, Collins R, Sleight P (1985) Beta blockade during and after myocardial infarction: An overview of the randomized trials. Prog Cardiovasc Dis 27: 335-371.

By the mid-1980s, the need to minimise the likelihood of being misled by the effects of biases and the play of chance in reviews of research evidence was being made evident in articles [10]–[14] and textbooks [15]. In 1988, regularly updated electronic publication of systematic reviews and meta-analyses, along with bibliographies of randomised trials, began in the perinatal field [16],[17]. This provided a model for the inauguration of the international Cochrane Collaboration in 1993 to prepare, maintain, and disseminate systematic reviews of the effects of health care interventions.

## Where Are We Now?

Despite this progress, the task keeps increasing in size and complexity. We still do not know exactly how many trials have been done. For a variety of reasons, a large proportion of trials have remained unpublished [18],[19]. Furthermore, many trials have been published in journals without being electronically indexed as trials, which makes them difficult to find. One of the first steps in being able to adequately review literature is that scientific contributions which predate digitalised information systems and trial indexing need to be “rediscovered and inserted into the memory system” [20]. Through the 1990s, to identify possible reports of controlled trials, the Cochrane Collaboration mobilised thousands of volunteers around the globe to comb the major databases, and to hand-search nondigitalised health literature, unpublished conference proceedings, and books. The result of this collaborative effort is the Cochrane Controlled Trials Register (CCTR) (now called the Cochrane Central Register of Controlled Trials).

**Figure 2:**
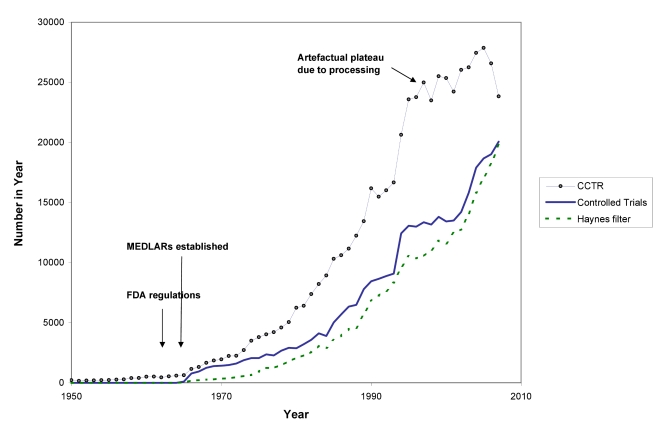
The number of published trials, 1950 to 2007. CCTR is the Cochrane Controlled Trials Registry; Haynes filter uses the “narrow” version of the Therapy filter in PubMed:ClinicalQueries; see Text S1.

The differences between the numbers of trial records in MEDLINE and CCTR (see Figure 2) have multiple causes. Both CCTR and MEDLINE often contain more than one record from a single study, and there are lags in adding new records to both databases. The NLM filters are probably not as efficient at excluding non-trials as are the methods used to compile CCTR. Furthermore, MEDLINE has more language restrictions than CCTR. In brief, there is still no single repository reliably showing the true number of randomised trials. Similar difficulties apply to trying to estimate the number of systematic reviews and health technology assessments (HTAs).

**Figure 3:**
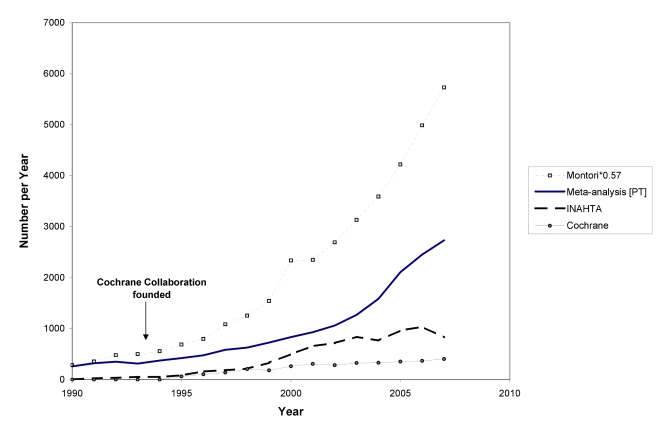
The number of systematic reviews in health care, 1990 to 2007. INAHTA is International Network of Agencies for Health Technology Assessment; the Montori systematic review filter is detailed in Text S1.

In Figures 2 and 3 we use a variety of data sources to estimate the numbers of trials and systematic reviews published from 1950 to the end of 2007 (see Text S1). The number of trials continues to rise: although the data from CCTR suggest some fluctuation in trial numbers in recent years, this may be misleading because the Cochrane Collaboration virtually halted additions to CCTR as it undertook a review and internal restructuring that lasted a couple of years.

Even though these figures must be seen as more illustrative than precise, multiple data sources tell the same story: astonishing growth has occurred in the number of reports of clinical trials since the middle of the 20^th^ century, and in reports of systematic reviews since the 1980s—and a plateau in growth has not yet been reached. With a median of perhaps 80 participants per trial, the number of people being enrolled in trials is likely to be more than 2,000,000 per year [21]. Prospective trial registration establishes a new genre of evidence repository: trials are registered in these databases at inception, theoretically enabling an overview of all published and unpublished trials.

In 2004, the International Committee of Medical Journal Editors (ICMJE, http://www.icmje.org/) announced that their journals would no longer publish trials that had not been prospectively registered [22]. Before this announcement, an average of 30 trials a week were being prospectively registered around the world. Once the journal editors' deadline came into force, more than 200 ongoing trials per week were being registered [23]. In 2007, the US Congress made detailed prospective trial registration legally mandatory [24]. As WHO's international clinical trials platform develops, it will become possible to generate a more realistic picture of how many trials are being done. This registry draws together standardised core data from all the trial registries meeting specified quality criteria. Registering full protocols and reporting trial results in these registries are the next frontiers.

## How Close Are We to Archie Cochrane's Goal?

**Figure 4:**
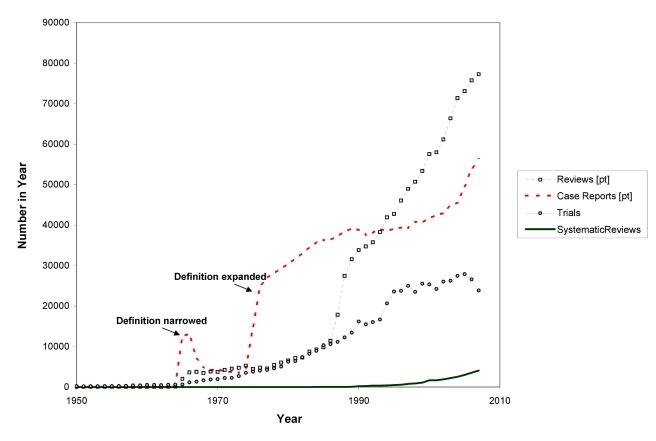
The rise in non-systematic reviews, case reports, trials, and systematic reviews, 1950 to 2007 (as identified in MEDLINE).

In 1986 and 1987, Goldschmidt and Mulrow showed how great the potential is for error in reviews of health literature that were not conducted systematically [9],[10]. Looking at data such as those in Figure 3 could provide the comforting illusion that systematic reviews have displaced other less reliable forms of information. However, as Figure 4 shows, this is far from the case. The growth has been even more remarkable in non-systematic (“narrative”) reviews and case reports. Journal publishing of non-systematic reviews, and the emergence of many journals whose sole product is non-systematic reviews, has far outstripped the growth of systematic reviews and HTAs, as impressive as the latter has been. And the number of case reports—which can also provide important new information such as adverse effects—is far higher than the number of trials or systematic reviews. Trials, systematic reviews, and HTAs have undoubtedly had major impacts, including on clinical guidelines: they are more likely to be cited and read than other study types [25]. However, the staple of medical literature synthesis remains the non-systematic narrative review.

Furthermore, we are a long way from having all relevant trials incorporated into good systematic reviews. The workload involved in producing reviews is increasing, and the bulk of systematic reviews are now many years out of date [26]. The median number of trials contained within individual systematic reviews has been variously estimated at between six and 16 (Cochrane reviews now include an average of over 12 trials per review [27],[28]; M Clarke, personal communication), but many reviews have covered much the same territory. Thus, in the 30 years since systematic reviews began in earnest, with around 15 years of intensified and large-scale reviewing effort, only a minority of trials have been assessed in systematic reviews. Given the triple constraint posed by the growth in trials, the increasing complexity of review methods, and current resources, Archie Cochrane's vision will not be achieved without some serious changes in course—in particular, with a greater concentration on Cochrane's use of the word “relevant”.

## Where to Now?

First, we need to prioritise effectively and reduce avoidable waste in the production and reporting of research evidence [29]. This has implications for trials as well as systematic reviews. Some funders and others will now not consider supporting a trial unless a systematic review has shown the trial to be necessary [30]. It is essential that this requirement be more widely adopted. And it is essential that reviews address questions that are relevant to patients, clinicians and policymakers.

Second, we may need to choose between elaborate reviews of a quarter of the questions clinicians and patients have or “leaner” reviews of most of what we want to know. The methodological standards for systematic reviewing have been increasing over time [28], and the evolution of standards in the Cochrane Collaboration and in HTA has been remarkable. The increase in steps and reporting required is reflected in the length of reviews. Early Cochrane reviews could typically be printed out in 10 or 20 pages, even when they incorporated several trials. Today, it is not unusual for a review by a health technology agency to run to several hundred pages. Often the reviews are longer than the combined length of the reports of all the included trials.

A contributing factor here is the increasing expectation for reviews to include study types other than randomised trials. This will often be essential for detecting less common adverse effects. However, the inclusion of all study types to answer all questions about the effects of treatments would not necessarily provide better quality information in every instance – while it would unquestionably increase the time and resource requirement for reviews. While it is vital that reviews are scientifically defensible, burdening those preparing them with excessive requirements could result in having valid answers to relatively few questions.

In particular, we need leaner and more efficient methods of staying up-to-date with the evidence. Using current methods, the Cochrane Collaboration has not been able to keep even half of its reviews up-to-date [31], and other organisations are in a similar predicament [32]. We need to develop innovative methods to reduce the labour of updating, and provide what clinicians and patients need: an assurance that a conclusion is not out of date, even if not every later trial is included within every analysis. It is also the responsibility of reviewer authors and journal editors to ensure that every new systematic review places itself clearly in context of other systematic reviews and HTAs. It will be to little avail to the average clinician, patient, and information provider, however, if the resulting knowledge is not comprehensible and openly accessible.

Finally, although more funding for evaluative clinical research internationally remains a priority, more international collaboration could result in better use being made of resources for systematic reviewing and HTAs. While multiple reviews on topics can provide a rounded picture of an area as well as a de facto form of updating when the reviews are conducted several years apart, there is also considerable duplication of review effort.

In November 2009, an international meeting in Cologne formed a new collaboration called “KEEP Up,” which will aim to harmonise updating standards and aggregate updating results. This should reduce the workload and enable organisations to be alerted when there are important shifts in evidence. Initiated and coordinated by the German Institute for Quality and Efficiency in Health Care (IQWiG) and involving key systematic reviewing and guidelines organisations such as the Cochrane Collaboration, Duodecim, the Scottish Intercollegiate Guidelines Network (SIGN), and the National Institute for Health and Clinical Excellence (NICE), this effort will provide a platform for tackling practical and methodological issues involved in keeping up-to-date.

There is nevertheless a risk that the increasing burdens placed on the methods of systematic reviewing could make the goal of keeping up-to-date with the knowledge won from trials recede ever more quickly into the distance. Perhaps one of the first questions we should ask whenever an additional process or more demanding methodology for systematic reviewing is proposed is this: Will this development serve or hinder our ability to better understand and communicate enough results from trials? In 1979, when Archie Cochrane argued that we needed critical summaries to keep up with the crucial knowledge those trials were generating, there were perhaps 14 trials a day being published. Thirty years later, it would be just as hard to keep up with the systematic reviews. Every day there are now 11 systematic reviews and 75 trials, and there are no signs of this slowing down: but there are still only 24 hours in a day.

## Supporting Information

Text S1 Search methods.
(0.03 MB DOC)

## Abbreviations

CCTR: Cochrane Central Register of Controlled Trials
FDA: US Food and Drug Administration
HTA: health technology assessment
IQWiG: Institute for Quality and Efficiency in Health Care
NICE: National Institute for Health and Clinical Excellence
NLM: US National Library of Medicine
SIGN: Scottish Intercollegiate Guidelines Network
