# Supplementary material for: Seventy-Five Trials and Eleven Systematic Reviews a Day: How Will We Ever Keep Up?
Source: PLoS Med. 2010 Sep 21;7(9):e1000326. doi: 10.1371/journal.pmed.1000326 (PMC2943439; doi:10.1371/journal.pmed.1000326)
Supplement: Text S1 — Search methods. (0.03 MB DOC) [file pmed.1000326.s001.doc]

# Search Methods

### Non-systematic reviews

For non-systematic reviews we used the Review publication type in PubMed.

### Case Reports

We used "Case Report" [Publication Type] limited to Humans. However according to the MEDLARS Technical Bulletins (http://www.nlm.nih.gov/pubs/techbull/tb.html), changes to coding in 1967 (to narrow the definition) and in 1975 (to re-expand the definition) create an anomalous dip in the apparent numbers of case reports.

### Systematic Reviews

1. The count of Cochrane reviews comes from quarterly data published on [www.cochrane.org](http://www.cochrane.org/) for up to the end of 2008.

2. The count on INAHTA (International Network of Agencies for Health Technology Assessment) comes for the INAHTA database at the Centre for Reviews and Dissemination.

3. We used two counts for MEDLINE. First we used the meta-analysis [publication type] limited to Humans. But this is known to miss many systematic reviews. So we also used one of the Montori filters [31]:

Medline[tiab] OR (systematic[tiab] AND review[tiab]) OR meta-analysis[ptyp], again limited to Humans. This filter has been assessed to have a sensitivity of 71% and precision of 57%. Given the 57% precision, we then multiplied the results by 0.57. Given the sensitivity of 71% this will underestimate the total number of systematic reviews. As we were interested in therapeutic reviews which are around 70% of all reviews [28], we kept this estimate as a reasonable one for therapeutic reviews.

### Trials

For trials we used three sources.

1. We used all trials in the Cochrane Central Register of Controlled Trials

2. For MEDLINE we used Pubmed and 3 publication types:

3. ("Controlled Clinical Trial " OR "Clinical Trial, Phase III " OR "Randomized Controlled Trial") limited to humans

4. And secondly the "narrow" version of the Clinical Queries filter for trials on PubMed.

All searches were done on April 18^th^ 2009.

**Supporting information**

**Text S1: Search Methods**

1. Boluyt N, Tjosvold L, Lefebvre C, Klassen TP, Offringa M. Usefulness of systematic review search strategies in finding child health systematic reviews in MEDLINE. Arch Pediatr Adolesc Med 2008;162(2):111-16.
2. Moher D, Tetzlaff J, Tricco AC, Sampson M, Altman DG. Epidemiology and reporting characteristics of systematic reviews. PLoS Medicine 2007; 4: e78.
